# Abatacept for the prevention of graft versus host disease in pediatric patients receiving 7/8 HLA-mismatched unrelated transplant for hematologic malignancies: a real-world analysis

**DOI:** 10.1038/s41409-023-02034-z

**Published:** 2023-08-14

**Authors:** Sharmila Raghunandan, Lev Gorfinkel, Brandi Bratrude, Yvonne Suessmuth, Kyle Hebert, Donna Neuberg, Kirsten M. Williams, Michelle L. Schoettler, Amelia A. Langston, Leslie S. Kean, Muna Qayed, John Horan, Benjamin K. Watkins

**Affiliations:** 1 Aflac Cancer and Blood Disorders Center, Children’s Healthcare of Atlanta, and Emory University, Atlanta, GA USA; 2 Dana-Farber Cancer Institute, Boston, MA USA; 3 Boston Children’s Hospital, Boston, MA USA; 4 Winship Cancer Institute, Emory University, Atlanta, GA USA

**Keywords:** Haematopoietic stem cells, Haematological cancer

## To the Editor:

Allogeneic hematopoietic cell transplant (HCT) offers a chance for cure in children with hematologic malignancies but is associated with significant morbidity. In the absence of a matched related or unrelated donor, a mismatched unrelated donor (MMUD) increases donor options, particularly for black, indigenous and people of color (BIPOC) patients, who have a less than 25% chance of having a matched unrelated donor (MUD) in the registry [1]. However, historically, MMUD HCTs have an increased risk for severe acute graft-versus-host-disease (aGVHD) resulting in increased treatment related mortality (TRM) and decreased overall survival (OS) when compared to matched related or unrelated donor HCTs [2–5] contributing to the lower survival of BIPOC patients receiving HCT [6].

Abatacept is a T-cell co-stimulation blockade agent [7] that was granted FDA approval for the prevention of aGVHD in patients receiving an unrelated donor (URD) HCT in part based on results from the “*Abatacept 2”* trial (ABA2, clinicaltrial.gov NCT01743131), a phase II trial that evaluated abatacept in addition to standard GVHD prophylaxis with a calcineurin inhibitor and methotrexate (CNI/MTX) for GVHD prevention in 8/8 MUD HCT and 7/8 MMUD HCT. In the ABA2 7/8 MMUD cohort, abatacept was associated with a significant reduction in grade 3-4 aGVHD compared with the matched CIBMTR cohort receiving CNI/MTX alone. The lower rates of grade 3-4 aGVHD translated into significant improvement in TRM and OS at 2 years [8].

The ABA2 trial and subsequent FDA approval paved the way for real world data that could validate these results and provide additional data on the potential benefit of abatacept. The ABA2 trial enrolled pediatric patients in the analysis, but outcomes were not reported separately for these patients. Given the lower risk of acute and chronic GVHD in pediatric patients [9], the translatability of the ABA2 results to this population would benefit from a focused analysis. Furthermore, patients treated on clinical trials may benefit from selection bias that can lead to improved outcomes compared to patients treated off study [10, 11]. The objective of this analysis was to explore the real-world impact of abatacept + CNI/MTX in pediatric patients not enrolled on ABA2.

## Methods

We conducted a retrospective analysis of outcomes for pediatric patients (<=22 years) at two centers who received a 7/8 MMUD HCT for hematologic malignancies and received GVHD prophylaxis with abatacept (4 doses, 10 mg/kg per dose, on days –1, + 5, + 14, and + 28) + CNI/MTX between January 2015 and December 2021. The analysis included pediatric patients transplanted at Children’s Healthcare of Atlanta and Boston Children’s Hospital. To compare these abatacept-treated patients to the most rigorous control, we included a comparison group from both centers who received an 8/8 HLA matched HCT for a hematologic malignancy, but who received CNI/MTX without abatacept (or other GVHD prophylaxis). Patients were excluded if they were on the ABA2 trial, not in remission at the time of HCT, received a prior HCT, or received GVHD prophylaxis other than CNI/MTX + abatacept.

Univariate analyses were performed to describe and compare outcomes between the groups. Grade II-IV aGVHD, grade III-IV aGVHD, moderate to severe chronic graft-versus-host disease (cGVHD), TRM and relapse are described as cumulative incidence estimates. Competing risks were relapse and death for the GVHD outcomes, relapse for TRM, and death in the absence of relapse for the relapse endpoint. Gray’s test was used to compare outcomes between the groups. OS, DFS, and grade III-IV acute GVHD-free, severe cGVHD-free, relapse-free survival (GRFS) are described as Kaplan-Meier survival probability estimates. The log-rank test was used to compare curves between the groups. All of the time-to-event endpoints are defined as the time from HCT until the event, the competing event, or until their last follow-up, whichever occurred first. Acute GVHD is analyzed at 180 days, while all other endpoints are analyzed at 1 year.

## Results

92 consecutive patients (27 7/8 MMUD patients who received CNI/MTX+abatacept and 65 comparator patients who received 8/8 MUD HCT with CNI/MTX) met inclusion criteria. Median follow-up was 741 days (range 182 - 2092 days). As expected, the 7/8 MMUD group had a greater number of patients from BIPOC compared to the 8/8 MUD group (52% vs 20%, *p* = 0.01). There were also differences in disease indication for transplant and disease stage between the two cohorts. AML (48%) was the most common indication for transplant in the 7/8 MMUD group whereas ALL (52%) was the most common in the 8/8 MUD group. Related to the differences in primary disease, the 7/8 group had a higher proportion of patients who had early disease stage at time of transplant (70% vs 28%). In both groups, nearly 95% of patients received a myeloablative conditioning regimen and were transplanted with a bone marrow graft (Table 1).

**Table 1:** Baseline Characteristics of Participants.

Baseline Characteristics	7/8 ABA	8/8 control	*p* value
	(*N* = 27)	(*N* = 65)	
Age at transplant, years			0.65
Median (range)	12 (1–22)	11 (<1–24)	
Age at transplant, grouped, no. (%)			0.16
0–9 years	13 (48)	26 (40)	
10–19 years	11 (41)	37 (57)	
20–24 years	3 (11)	2 (3)	
Sex, no. (%)			0.07
Male	18 (67)	29 (45)	
Female	9 (33)	36 (55)	
Race, no. (%)			0.01
White	13 (48)	47 (72)	
Black/African American	12 (44)	10 (15)	
Asian	2 (7)	3 (5)	
Unknown	0	5 (8)	
Ethnicity, no. (%)			0.68
Hispanic or Latino	7 (26)	11 (17)	
Not Hispanic or Latino	19 (70)	51 (78)	
Unknown	1 (4)	3 (5)	
Primary disease for transplant, no. (%)			0.05
AML	13 (48)	20 (31)	
ALL	7 (26)	34 (52)	
CML	3 (11)	1 (2)	
MDS	3 (11)	6 (9)	
Other	1 (4)	4 (6)	
Disease stage, no. (%)			0.0002
Early	19 (70)	18 (28)	
Intermediate	6 (22)	43 (66)	
Advanced	2 (7)	3 (5)	
Not categorized	0	1 (2)	
Conditioning regimen, no. (%)			0.11
Busulfan/Fludarabine	9 (33)	11 (17)	
Busulfan/Cyclophosphamide	7 (26)	13 (20)	
Total Body Irradiation/Cyclophosphamide	7 (26)	35 (54)	
Fludarabine/Melphalan	2 (7)	3 (5)	
Clofarabine/Busulfan/Fludarabine	2 (7)	3 (5)	
Conditioning intensity, no. (%)			0.63
Myeloablative	25 (93)	62 (95)	
Reduced Intensity	2 (7)	3 (5)	
Graft type, no. (%)			0.63
Bone marrow	25 (93)	62 (95)	
Peripheral blood	2 (7)	3 (5)	
Median follow-up of survivors, months			0.87
Median (range)	24 (6–66)	25 (7–69)	

*AML* acute myelogenous leukemia, *ALL* acute lymphatic leukemia, *CML* chronic myelogenous leukemia, *MDS* myelodysplastic syndrome.

**Fig. 1:**
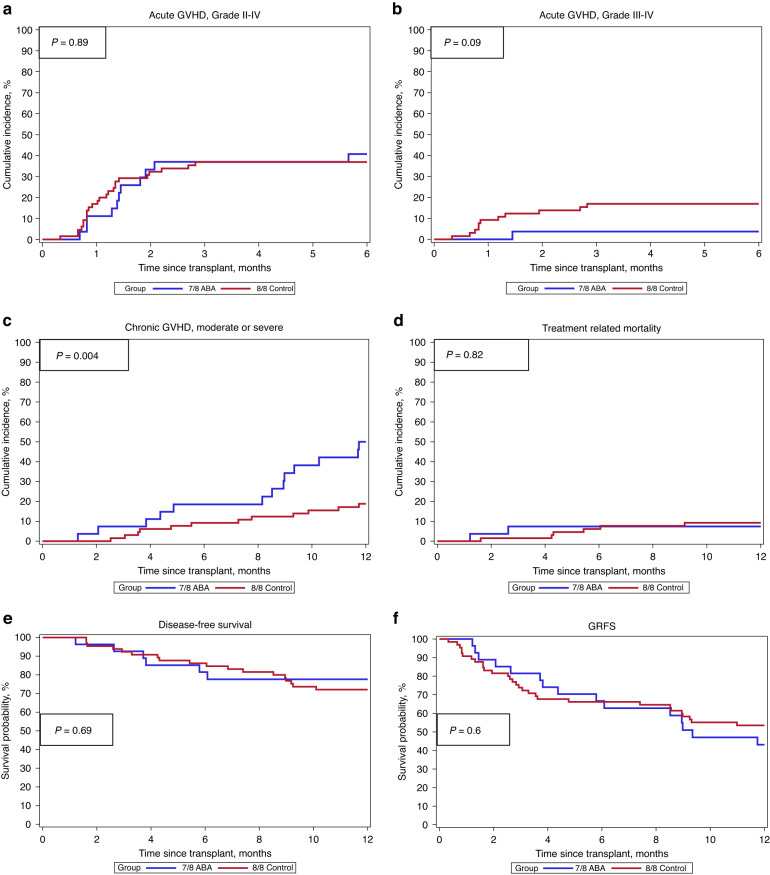
Cumulative incidence of GVHD and Treatment Related Mortality and Kaplan-Meier plots of Survival Outcomes. **a** Cumulative incidence of Grade 2–4 aGVHD. **b** Cumulative incidence of Grade 3-4 aGVHD. **c** Cumulative incidence of Moderate-Severe cGVHD. **d** Cumulative incidence of Treatment Related Mortality. **e** Kaplan-Meier plot of Disease-Free Survival. **f** Kaplan-Meier plot of grade III-IV aGVHD-free, severe cGVHD-free, relapse-free survival. aGVHD acute graft-versus-host disease, cGVHD chronic graft-versus-host-disease.

Cumulative incidence of grade II-IV aGVHD was similar for the 7/8 CNI/MTX+abatacept MMUD and 8/8 CNI/MTX MUD groups (41% vs 37%, *p* = 0.89). Grade III-IV aGVHD was non-significantly lower in the 7/8 CNI/MTX+abatacept MMUD group compared to the 8/8 CNI/MTX MUD group (4% vs. 17%, p = 0.09). Cumulative incidence of moderate-severe cGVHD was higher in the 7/8 CNI/MTX+abatacept MMUD group compared to the 8/8 CNI/MTX MUD group (50% vs 19%, *p* = 0.004). Rates of TRM (7% vs 9%, *p* = 0.82), relapse (15% vs 19%, *p* = 0.72), overall survival (85% for both groups, *p* = 0.99), disease-free survival (DFS) (78% vs 72%, *p* = 0.69) and GRFS (43% vs 54%, *p* = 0.6) at 1 year were similar between the two groups (Fig. 1). Neither cohort had end-organ CMV disease nor post-transplant lymphoproliferative disease.

## Conclusion

Our real-world results using abatacept prophylaxis for 7/8 MMUD HCT mirror the results from ABA2 trial with low incidences of grade III-IV aGVHD and TRM and encouraging DFS. The 7/8 MMUD patients who received abatacept had similar outcomes to 8/8 MUD patients without abatacept within the first year from HCT, similar to what we have previously reported for the ABA2 trial [12]. These results are promising given the increased HLA mismatch in the abatacept group in this analysis. Similar to results from the ABA2 trial, cGVHD rates did not appear to be impacted with the 4 dose abatacept regimen, and an on-going study is evaluating extended dosing of abatacept to address this issue (ABA3, clinicaltrial.gov #NCT04380740). Despite the higher rates of moderate-severe cGVHD in the 7/8 MMUD group, GRFS was similar between the two groups, likely secondary to the rates of grade III-IV aGVHD that trended lower in the 7/8 CNI/MTX+abatacept MMUD group. There are some limitations of this analysis, most notably that it was a retrospective study with a relatively small sample size. It is also important to highlight that there were differences in disease indication for transplant and disease stage between the two cohorts that could have contributed to improved outcomes in the 7/8 group. Finally, the results of this analysis are based on two pediatric centers and therefore it’s generalizability to all pediatric patients could be limited. Larger and multi-center real-world studies will be needed to validate results from our analysis.

In summary, these real-world results suggest that CNI/MTX+abatacept immunoprophylaxis for pediatric patients receiving a 7/8 MMUD HCT results in survival outcomes that are similar compared to pediatric patients receiving more highly matched transplants with CNI/MTX prophylaxis. By mitigating the risks of mismatching, abatacept may help improve outcomes for BIPOC patients who are more often transplanted with an MMUD HCT.

## Data Availability

Data collected for this study will be made available with publication to anyone who wishes to access the data for any purpose; please contact the corresponding author: sraghu2@emory.edu.
